# Evaluation of the Effectiveness of Outdoor Fitness Equipment Intervention in Achieving Fitness Goals for Seniors

**DOI:** 10.3390/ijerph182312508

**Published:** 2021-11-27

**Authors:** Hsueh-Wen Chow, Kun-Tang Chang, I-Yao Fang

**Affiliations:** 1 Graduate Institute of Physical Education, Health & Leisure Studies, National Cheng Kung University, Tainan 70101, Taiwan; tedted33333@gmail.com; 2 Center of Physical Education, Southern Taiwan University of Science and Technology, Tainan 71005, Taiwan; fyy125@gmail.com

**Keywords:** fitness zone, endurance training, resistance training, flexibility, balance, range of motion, park

## Abstract

Despite the popularity of outdoor fitness equipment (OFE) in public spaces with the aim of increasing physical activity (PA), very little research has been conducted to investigate the effectiveness of the equipment’s use, especially for achieving the target fitness goal. This study designed an OFE training protocol incorporating four modalities of PA: endurance (air walker and ski machine), resistance (rowing machine and bonny rider), flexibility (arm stretch and shoulder wheel), and balance exercise (waist twister). Seniors who completed the protocol demonstrated that for endurance training, participants were at moderate intensity from heart rate and perceived exertion, while significantly improving cardiac endurance in the two min step test. For resistance/flexibility/balance interventions, only the handgrip strength test, the 30 s chair stand test, the right-hand shoulder flexion test, the right-hand shoulder horizontal abduction test, the single-leg stance test, and the eyes open foam surface clinical test for sensory balance were significant, using a repeated measure analysis of variance. The results demonstrated that older adults aim for the OFE target for specific fitness goals can reach moderate intensity and improve their cardiorespiratory endurance. Yet, results for resistance/flexibility/balance are inconclusive. More studies are needed to carefully assess the effectiveness of using OFE.

## 1. Introduction

Physical inactivity is a major health challenge, especially for seniors [1,2]. Parks and recreation professionals are recognized as essential contributors to active aging since built environments in recreational sites and parks are the preferred venues for most seniors to increase their physical activity (PA) [3,4]. They are more effective than other programs toward sustainability and reaching a number of populations [4,5]. Parks usually provide easy access in their geographical proximity and tend to be equipped with exercise facilities such as swimming pools and ball courts [6]. The current literature addresses the upgrading of open green spaces or built environments to promote PA as its potential to reach a broader and more sustainable base of citizens [5,7,8,9,10,11,12,13,14].

Many parks worldwide installed outdoor fitness equipment (OFE) in parks/open spaces to attract people [15,16,17,18,19]. OFE, or outdoor gyms, fitness zone, have become popular in numerous green spaces and built environments [20,21,22,23]. These facilities are modified configurations of conventional indoor gym equipment to use in the outdoor environment. OFE offers the local neighbourhood free access to fitness training [24,25], beneficial to those in low-income areas or those who cannot afford expensive gym memberships [15].

A growing body of research has investigated the use of OFE and its benefits: it attracts several visitors to parks and encourages them to be active [15,17,26]. Studies determined that the installation of OFE attracted new visitors to parks and was cost-effective as well [15,27,28]. The installation of OFE also contributes to city scenery or landscape, and improves safety perceptions [15,29]. There are unique benefits linked to OFE use not limited to physical health, e.g., fitness, muscle strength, and weight loss, but to psychological health and social connections with family/friends and other users in natural outdoor spaces [27,29,30,31]. Despite OFE expansion, there are many associated issues such as safety concerns, lack of maintenance, not fit for purpose design, falling short of promotion strategies, and absence of instruction for proper use [31,32,33,34,35].

Specifically, there is little research to assess the use of the equipment [36]. Many issues surfaced with OFE: lack of correct use [25,37] and no guidelines on duration and intensities [38,39]. The scientific evidence is minimal; some studies claim that OFE can achieve moderate to vigorous PA [15,17], while others found that it provides only light intensity [39]. Previous studies found that users did not exercise long enough to achieve health benefits. Observation studies found users operate OFE for less than 10 min [40,41]. This conflicts with the American College of Sports Medicine (ACSM) and the American Heart Association (AHA) guidelines, in which older adults are advised to engage in 30 min of moderate-intensity PA for five days (150 min per week). Each activity should be at least 10 min [2,42]. Thus, the benefits of OFE in achieving a higher level of PA to improve health is questionable.

Although OFE users may combine a workout with other activities, such as walking/hiking/tai-chi, etc., proper guidelines for OFE are necessary to achieve the recommended PA during a park visit. So far, the scientific evidence to support OFE’s effectiveness presents mixed results because previous studies examined the overall OFE program without examining the different types of goals for cardiorespiratory fitness, muscle strength, balance, and flexibility. [17,43,44,45].

With advancing age, structural and functional deterioration occurs for most individuals. Aging is associated with several physiologic changes, such as reduced functional capacity and changing body composition, etc. To preserve optimal physical function and quality of life, the ACSM and the AHA issued exercise and PA guidelines for older adults [2,42]. The guidelines incorporate several modalities of PA, including endurance exercise, resistance exercise, flexibility exercise, and balance exercise. For endurance exercise, older adults should engage in a minimum of 30 min of moderate-intensity PA for five days and a total of 150 min per week. Alternatively, older adults could involve vigorous-intensity activities for a total of 75 min per week. The duration for each activity would be at least 10 min in each bout; for resistance exercise, older adults should conduct a weight training program at least twice a week to reach an intensity between moderate (5–6) and vigorous (7–8) on a 0–10 scale. Resistance training should involve major muscle groups of 8–10 repetitions each. Seniors should also engage in flexibility exercise at least twice a week with moderate (5–6) intensity. For unspecific guidelines for balance exercises, ACSM suggests progressive steps, such as reducing the base of support, reducing sensory input, and stressing postural muscle groups to increase difficulty of balance training [42].

Despite the rapid expansion of parks with OFE, until now, few studies examined the actual use and effectiveness of OFE. Many studies highlight the need for guidance on appropriate equipment use [16,17,25,30,32,34,40,46]. Hence, this study aims to assess the effectiveness of OFE for seniors. It was hypothesized that the two 12- week OFE training intervention would positively influence the four domains of fitness levels, cardiorespiratory, muscle strength, flexibility, and balance, of the study population by objective and functional fitness measurement. These issues deserve further investigation to optimize rising investment in installing OFE.

## 2. Materials and Methods

### 2.1. Subjects

Research flyers were distributed at parks and community bulletin boards to recruit seniors to take part in this experiment. Inclusion criteria for participation were: (a) aged 60 or above, (b) healthy to the degree that participation in exercise testing and a related exercise program would not exacerbate any existing symptomology, (c) personal physician clearance for participation, (d) adequate mental and cognitive status, and (e) willingness to participate in 12 weeks of training. We used the Physical Activity Readiness Questionnaire (PARQ+) to determine if individuals were suitable to participate in the study [47].

### 2.2. Ethics Approval

The study was conducted according to the guidelines of the Declaration of Helsinki and approved by the National Cheng Kung University Human Research Ethics Committee (NCKU HREC-E-107-382-2). The research team hosted an information session before two interventions. The information sessions informed participants that their involvement was voluntary and that they had a right to withdraw at any time. Informed consent was obtained. 

### 2.3. Equipment and Interventions

The training protocol for each type of exercise and its designated assessments are presented in Table 1 and detailed description in the following sessions. All assessments follow standards for testing and training, which the AHA recommends ensuring participant safety [48]. The research team evaluated the fit-for-purpose of each OFE and selected appropriate equipment to represent endurance exercise, resistance exercise, flexibility exercise, and balance exercise, respectively [38]. Next, based on ACSM guidelines and instructions from several manufacturers, we designed appropriate OFE protocols. Each training started with a warm-up session, followed by main OFE activity sessions, concluding with a cool-down session. There were two intervention phases, each lasting for 12 weeks. The first phases focused on cardiorespiratory training for five days per week, and the second phase targeted muscle strength, balance, and flexibility for two sessions per week. During the intervention, a certified fitness trainer for seniors was presented on-site and demonstrated how to correctly use OFE machines to ensure that all participants used them correctly. The trainer verified participant compliance, timed each intervention, and answered their questions. We examined performance objectively after using each fit-for-purpose equipment. A small monetary reward ($80) for completing each 12-week intervention and benefits of personal fitness test reports encouraged seniors to complete the program.

#### 2.3.1. Phase 1: Cardiorespiratory Endurance Training

Based on the classification of fit-for-purpose of OFE [38], the air walker and ski machine were the most popular cardiorespiratory endurance training to develop cardiovascular function. Participants took part in the air walker and ski machine cardio training sessions five days a week for 12 weeks. Each session began with a 5 min warm-up and two 20 min activity sessions at a rate of 60 steps per minute. Sessions concluded with a 5 min cool-down period, while a 3 min break between the two main activities allowed participants to rest.

##### Assessment

The assessment includes heart rate and self-reports of perceived exertion. Heart rate is an important indicator to monitor cardio endurance training, as it could objectively reflect the physiological response of training intensity [49,50]. Therefore, for participants’ heart rate during the 12-week aerobic OFE exercise, they wore the POLAR H10 (Polar Electro Oy, Kempele, Finland) heart rate strap on their chest to monitor heart rate during one exercise session every week. Each participant’s second-by-second HR data from Polar H10 during the cardiorespiratory endurance training session was first obtained by a cardio training app (Cardio Training, Angelmarcos, Brantford, ON, Canada) once every week and exported as CSV files. In addition, participants were asked to report the 10-point Borg Rating of Perceived Exertion Scale (RPE) [51] for a subjective assessment of exercise intensities. The 2 min step test measures the number of times seniors lift their knees to a height midway between their patella and iliac crest in a standing position for two minutes [52]. All assessments in phases 1 and 2 were assessed at baseline (week 0), midterm (week 6), and intervention completion (week 12).

#### 2.3.2. Phase 2: Muscle Strength, Balance, and Flexibility Training

During the second phase of intervention, participants were prompted to engage in the sessions two days per week in the presence of a fitness trainer. The exercise program encompassed set exercises that utilized five pieces of OFE to match the fit-for-purpose training, respectively. Each session started after a 5 min warm-up, as participants operated five types of equipment for different training purposes. They decided on the order of equipment used. However, equipment belong to the same fitness training goal was required to be used before moving on to the next type. 

##### Strength Training

Strength training is a promising intervention to reverse the deterioration of muscle strength associated with aging [53]. The principle of strength training for older adults is progressive training methods to gradually increase the external force or repetitions for muscle adaptation. A rowing machine and bonny rider were adopted for strength training. Unlike the conventional indoor resistant training machine that allows adjustment for different weights, the use of OFE only operate the machine against their own body weight. As such, the resistant level is consistent. In order to follow ACSM guidelines of progressive training [42,54], this study’s intervention protocol gradually increases the number of repetitions for each training set. The participants only perform eight repetitions of an exercise for three sets during the first six weeks of strength training, and increase to 12 repetitions for three sets in the second six weeks of training.

##### Assessment

Grip strength is an indicator of overall physical strength and health [55] as study demonstrates a positive significant association between grip strength and global muscle strength for older adults [56]. The feature of easy to measure, non-invasive, and good validity and reliability, allow measuring grip strength to be widely used in many clinical and field studies for seniors [49,57]. Participants performed the test with their dominant hand in two attempts. The highest value was used to determine maximal grip strength, an individual’s maximum strength capacity of the muscle group they can lift just once, and to represent their muscle strength. We also assessed participants’ change of muscle mass, body fat percentage, and skeletal muscle mass index (SMI) with the body composition analyser using bioelectrical impedance analysis (InBody 270, Biospace, Cerritos, CA, USA), which has been tested with excellent validity and reliability [58]. The subjects were required to stand barefoot on a scale for a few seconds. For the functional test, we adopted the 30-s chair stand test, in which we timed the number of participants from a seated position to full stands in 30 s, to assess seniors’ lower body strength [52]. 

##### Flexibility Training

The arm stretch and shoulder wheel were identified for flexibility training. The participants followed the tempo at 60 bpm while operating the shoulder wheel, holding static stretches unilaterally for 30-s with the arm stretch. Each set of flexibility training exercises lasted for five minutes.

##### Assessment

As these two pieces of equipment were designed for shoulder flexibility, we used goniometry (measured in degrees) to assess the shoulder’s range of motion (ROM) while participants were in a supine position. Measures included shoulder flexion, shoulder extension, shoulder horizontal abduction, shoulder horizontal adduction, shoulder internal rotation, and shoulder external rotation [59]. In addition, the back-scratch test, which measures how close two hands can reach behind the back is also an indication of general shoulder ROM from the functional fitness test [52]. 

##### Balance Training

The waist twister (rotator) was selected for balance training. Participants were asked to stand on the OFE station and rotate clockwise and counter-clockwise at a speed of 60 bpm during the exercise. ACSM suggests progressively increasing the difficulty of balance tasks, such as reducing the base of support, changing the centre of gravity, stressing postural muscle groups, or reducing sensory input; all are beneficial for balance training [42]. Hence, the training protocol was designed to increase the difficulty every four weeks. For a total of 10 min of balance training, the first four weeks, participants could stand on the platform with two feet for 10 min. During weeks 5–8, participants were asked to stand with only one foot for 2 min, and for weeks 9–12, participants stood with only one foot for 4 min; in this way, the instability of the base was gradually increased. 

##### Assessment

The Romberg test (Romberg, 1853) is a standard method of static balance testing [60]. The Clinical Test of Sensory Integration of Balance (CTSIB), one Romberg test, was used to measure elderly balance in this experiment. The CTSIB has four scenarios: Eyes Open Firm Surface, Eyes Closed Firm Surface, Eyes Open Foam Surface, and Eyes Closed Foam Surface. The Biodex Biosway Balance System (BBS; Biodex Inc., Shirley, NY, USA), a multiaxial device that objectively measures and records an individual’s ability to stabilize the involved joint under dynamic stress, which is used to evaluate postural balance. The Biodex Biosway has an intraclass correlation coefficient of 0.81 with a 95% confidence interval [61]. The higher number indicates one’s level of instability during the test. 

The researcher also measures static and dynamic balance for participants. The static balance was assessed by the one-leg standing test [62]. Subjects start with both legs standing on the ground comfortably and lifting one leg to the other ankle with the eyes open and arms by the side. Time with one foot on the ground was measured: a longer time indicated better balance ability [63]. For dynamic balance, participants performed the 8-foot up-and-go test, the duration of a subject seated initially, who then walked as fast as possible to and around a cone 8 feet away and returned to sit down [52].

### 2.4. Statistical Analyses

The researcher uses SPSS 28 (IBM Corp.) to perform a statistical analysis. We first checked the assumption of normality of the data in all the treatment conditions. The participants’ demographic characteristics (age, gender, health status, OFE experiences) and all assessment information at various time points (baseline, middle, post-intervention) were shown with descriptive statistics (mean ± standard deviation). In order to achieve the research’s goal, three repeated-measured multivariate analysis of variance (MANOVA) was conducted to test for change over time in seniors’ muscle strength, flexibility, and balance. If the results are significant, post hoc one-way repeated measure analysis of variance (Repeated measure ANOVA) was subsequently performed to examine changes for corresponding specific fitness measurement among pre-midterm-post training. If the sphericity assumption is violated, a Greenhouse–Geisser correction was applied [64]. If the main ANOVA is significant, this means that there is a difference between at least two time points. The post hoc pairwise comparisons contain multiple paired t-tests with a Bonferroni correction was checked to keep the Type I error at 5% overall. All significance was accepted at *p* < 0.05. 

## 3. Results

### 3.1. Participant Characteristics

A total of 20 seniors (six males and 14 females) completed the first phases of the study. In the second phase of recruitment, the 20 participants from phrase one expressed their interests to continue participating the phase 2 experiment and another nine more seniors joined for a total of 29 seniors (11 male and 18 female) and completed the second phase of the study. Three participants did not take the balance and body composition mid-test due to personal scheduling issues. The average age was 66.0 ± 4.3 (range: 60–76), and the average BMI was 23.4 ± 3.5 (range: 16.9–30.2 kg/m2). In terms of behaviour, most senior citizens (93%) have regular exercise routines. Seven had never visited the parks, while others engaged in light intensity PA in the park, such as stretching (37.9%) or walking (27.6%). Only 10 participants had used OFE before.

### 3.2. Changes in Cardiorespiratory Endurance Training

**Figure 1:**
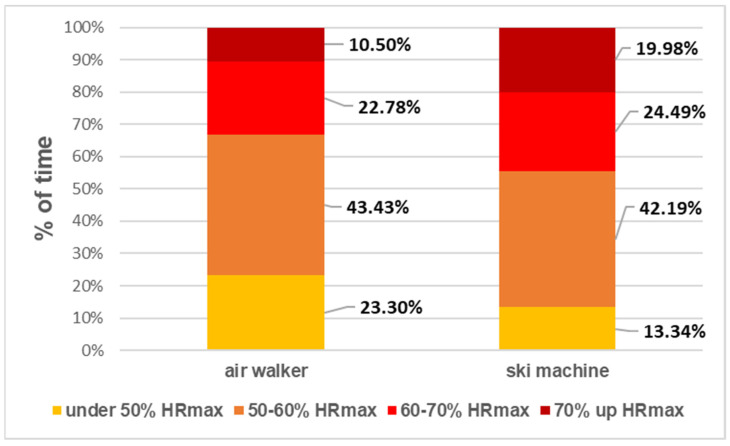
Heart rate zones from all participants while operating the air walker and ski machine for 12 weeks.

Heart rate (HR) is valuable physiology information representing the intensity or training load of an exercise. The average HR of older adults during the twelve-week exercise was 90.59 ± 16.86 bpm. We evaluated the individual internal training load by expressing the HR record as the percentage estimated by the participants’ maximum HR (HRmax) and categorized them into four target HR zones. Figure 1 illustrates the percentage of time that all participants’ HR fell into each HR zone. For example, participants’ HR was about 10.50% of the time above 70% HR_max_ and 22.78% of the time between 60–70% HRmax in the air walker session. In the ski machine session, the participants’ HR reached about 19.98% of the time above 70% HR_max_. During the operation of OFE, most participants are able to keep their HR above 50% HRmax. Participants showed significant differences while operating the air walker versus the ski machine, in terms of their HR for different heart rate zones (X_2_ (3.58) = 16,526.49, *p* < 0.001). The time the participants’ HR was above 50% HR_max_, with the HR during the ski machine session higher than that of the air walker (Ski: 86.66%, Air walker: 76.71%) (Figure 1).

The subjective assessment of exercise intensities in RPE ranged from 1 to 7 with a mean of 3.20 ± 1.71 (on a 10-point scale). This indicates that older adults perceived using the air walker and ski machine as light to moderate intensity [51]. 

Participants performed a 2 min step test at week 0, 6, and 12 to assess changes in cardiorespiratory endurance, revealing a significant difference between the pre-test (96.60 ± 11.72), mid-test (106.90 ± 13.77), and post-test (115.70 ± 13.71) (F_(2,38)_ = 90.94, *p* < 0.001). 

### 3.3. Changes in Muscle Strength

Participant muscle strength changes were measured from three perspectives: body composition test, handgrip strength test, and 30-s chair stand test. Results from the repeated measure MANOVA showed that significant multivariate effects for time (Wilks’ Lambda = 0.44, F(_10,92_) = 4.67, η_p_^2^ = 0.337, *p* < 0.001). In the body composition test, the participants’ muscle mass, body fat rate, and SMI did not reach a significant difference after intervention. However, participants’ handgrip strength increased from 24.52 ± 1.31 kg at baseline to 27.97 ± 1.61 kg afterwards. Participants’ 30-s chair stand test also improved from 20.72 ± 1.11 times at baseline to 24.35 ± 1.17 at the end. There were significant differences between the handgrip strength test (F_(2,56)_ = 8.95, *p* < 0.001) and the 30-s chair stand test (F_(2,56)_ =11.09, *p* < 0.001). There is a significant increase in participants’ muscle strength performance after the intervention.

### 3.4. Changes in Flexibility

The flexibility changes were measured from participants’ shoulder ROM and the back-scratch test. The omnibus results showed a significant main effect for time (Wilks’ Lambda = 0.362, F(_30,84_) = 1.852, η_p_^2^ = 0.398, *p* = 0.015). From repeated measure ANOVA results, among 14 shoulder ROMs measured, there were only significant differences in two shoulder motions, which included: right-hand shoulder flexion (with the Greenhouse–Geisser correction, F_(1_._34, 37_._64)_ = 4.86, *p* = 0.02), and right-hand shoulder horizontal abduction (F_(2,56)_ = 7.03, *p* < 0.001). For the back-scratch test, there were no significant differences during the intervention.

### 3.5. Changes in Balance

The balance test in this study included dynamic balance, static balance, and CTSIB. For the four test conditions, from CTSIB testing posture stability, the higher number indicated a more unsteady individual throughout the test. Repeated measure MANOVA revealed significant main effect for time (Wilks’ Lamda = 0.588, F(_12,90_) = 2.283, η_p_^2^ = 0.233, *p* = 0.014), with univariate statistics indicating a significant increase in single leg stance test and eyes open foam surface. The results from repeated measure ANOVA show that although participants performed better at the end of the intervention compared to baseline, only the test for the eyes open foam surface condition was statistically significantly increased (F_(2,50)_ = 4.88, *p* < 0.001). We also observed significant differences in the single-leg stance test (with the Greenhouse–Geisser correction, F_(1_._58,44_._36)_ = 7.47, *p* = 0.003). For other tests, no significant changes were observed. The results from series of repeated measure ANOVA are shown in Table 2.

## 4. Discussion

### 4.1. The Air Walker and Ski Machine OFE Training Achieves Moderate Cardiorespiratory Training

Endurance and aerobic exercise training can improve cardiorespiratory endurance of older adults and provide beneficial protection for similar aging in later life [65]. This study confirmed that seniors operating the air walker and ski machine achieved moderate intensity of PA via the objective HR data and subjective perceived exertions. This is consistent with earlier studies, while using the metabolic system, suggesting that the metabolic equivalent (MET) for operating the air walker ranges from 2.81 METs to 3.55 METs, while the ski machine ranges from 3.02 METs to 4.05 METs [39].

However, earlier studies only measured short periods (three minutes) [39]. This study asked participants to follow ACSM and AHA’s guidelines for a moderate-intensity aerobic activity for a minimum of 30 min, five days per week over 12 weeks. The tempo is slightly reduced from earlier studies (80/100/120 bmp to 60 bpm) to prevent seniors from feeling fatigued while operating the equipment for an extended time (40 min each session). The HR data and RPE confirmed that this tempo helped older adults achieve moderate intensity during most exercise sessions. Participants also improved their cardio functional fitness, seen in the 2 min step test.

### 4.2. The Rowing Machine and Bonny Rider OFE Training Cannot Change Body Composition but Can Improve Both Upper and Lower Limb Muscle Strength

Loss of muscle mass with aging is associated with older adults’ muscle strength and deterioration of physical function. In this study, objective body composition data and performance-based tests were obtained to examine the effectiveness of OFE resistance training for muscle strength. Although the overall strength is improved significantly, none of the data from body composition changed significantly. This may be attributed to many OFE machines, unlike conventional resistance training equipment in the gym, which cannot be adjusted for different weights to increase resistance levels [39,44]. In this study, participants operated the rowing machine and bonny rider only against their own body weight. The exercise intervention protocol modifies the number of repetitions to increase the resistance training load. Thus, the effectiveness of adding weight or progressively increasing resistance in strength training may not be optimal. 

Despite this, there is significant improvement from the participants’ handgrip strength test and the 30 s chair stand test. Hand grip strength is a critical predictor of older adults’ health status, such as quality of life, number of hospitalizations, functional capacities, and death [66]. In this study, participants must use their upper limb muscles to hold the handle of the rowing machine and bonny rider, which can train handgrip strength. In addition, the pull from the handle of the bonny rider involved the lower limb muscles to complete the entire movement. The 30 s chair stand test, examining lower limb muscle strength, also showed significant improvement.

### 4.3. Arm Stretch and Shoulder Wheel OFE Improve Partial Shoulder Range of Motion

Flexibility is a vital function that many older adults must maintain to avoid physical impairments or reduced daily living activities. Stretching exercises are critical to increase flexibility. After training of the arm stretch and shoulder wheel OFE, although the overall main effect is significant, only 2 out of 14 shoulder ROM measurements had significantly improved. Several reasons explain these results. First, the arm stretch, and shoulder wheel movement are not a full shoulder ROM. Unlike the professional shoulder wheel in physical therapy clinics, with a variety of arc of motions and resistance levels, the arc of motion is limited, and the resistance is fixed in this study. Second, the literature suggests that adding weight to stretch training is an effective way to improve flexibility for older adults [67]. However, we cannot add weight for the arm stretch and shoulder wheel in OFE. Although participants operate the arm stretch and shoulder wheel with both hands, only right-handed shoulder abduction and flexion ROM showed significant improvement.

### 4.4. Single Leg Standing Training on the Waist Twister OFE Enhances Seniors’ Static Balance Tests

Balance training is beneficial to prevent falls, critical to keep seniors independent, and maintain good quality of life. In this study, with the waist twister OFE, we reduced the time of base support to challenge the alignment of the body central to gravity [68]. This training seemed to be effective, as the participants’ single-leg stance improved significantly after the intervention. The results demonstrated significant improvement from participants’ eye open foam surface test, but not eye closed tests; or on the eye open firm surface test, which might be attributed to the training of incorporating an unstable base of support, this was not from visual sensory input.

## 5. Conclusions

Earlier intervention studies did not specify different types of OFE and their impact. This study examined four types of OFE and followed ACSM guidelines to design the specific fit-for-purpose exercise protocol and investigate effectiveness with objective measures and senior functional tests. 

The results show that participants were at moderate intensity with the air-walker and ski machine, which significantly improved cardiac endurance in the 2 min step test over the course of the 12-week intervention. In terms of resistance training, the bonny rider and rowing machine helped participants significantly increase their handgrip strength and the 30 s chair stand test, but this did not apply to body composition parameters. There is a significant increase in participants’ hand and lower limb muscle strength after the intervention. Regarding flexibility, evidence from this study suggests that there is minimal improvement in participants’ shoulder ROM and flexibility after the arm stretch and shoulder wheel flexibility training. As for the balance aspect, while increasing the challenge at the base of support on the waist twister, participants performed significantly better on the static balance test and one postural stability test, but their performance did not improve on the dynamic balance test, as well as on some postural stability tests. 

Overall, this study yields inconclusive evidence of the OFE regarding seniors’ fitness tests and functional performance. This might be attributed to the fact that, although many OFE programs are modified from gym equipment or physical therapy devices, they do not compare to exact functionality. Many OFE exercises cannot add weight to increase resistance level or adjust the length or width to fit different individuals’ heights, fitness levels, or therapeutic needs. Most senior participants from this study have good/fair health status, so the 12-week intervention may not have gained noticeable improvement in terms of fitness and functional performance. 

The generalizability of our results is subject to certain limitations. First, the OFE may not be the same as those in different regions: different OFE manufacturing design and equipment slightly differ, despite a similar appearance [23]. Second, the interventions were conducted in a park to reflect free-living experience when seniors use OFE. As such, some parameters can be affected by environmental issues. For example, a person’s HR is influenced by several individual and environmental factors, including emotions and stress, lifestyles, nutrition and water intake, medications, and environmental temperature and humidity [69,70]. This study strives to control for these factors by instructing participants to be consistent with lifestyle and mood, but external stimuli are sometimes unavoidable. Third, as it is a considerable commitment for seniors to participate in a total of 24 weeks (2 phases with each lasting 12 weeks) of OFE exercise interventions, with limited OFE available in the park to schedule more participants in the training, it is inevitable to result in a small sample with no control group, which can limit generalizability. Fourth, given that the seniors in this study have more regular exercise routines compared to their general counterparts, the effects of the intervention on fitness performance could be limited [44]. Finally, as we incorporate resistance, flexibility, and balance training in the same phase of the intervention, some training effects may have carried from one aspect to another. The improvement of a participant’s shoulder ROM may not have only resulted from flexibility training but also from strength training. 

Despite these limitations, this study offers valuable insight to understanding the effectiveness of each specific fit-for-purpose OFE. Findings of this study can provide evidence-based data and substantiate instruments for OFE manufacturers, fitness trainers, public health experts, local municipal authorities, and public health agencies to evaluate the effectiveness of OFE for active lifestyle programs and concomitant environments. Fitness trainers who adopt OFE as their training site and public health officials who promote active OFE environment can benefit from the evidence-based data from this study.

More work may determine OFE’s effectiveness. There are only seven OFE exercises in this intervention, while several other types can be examined. The design of the OFE exercise protocol could be expanded. For example, for balance training, we only incorporate a changed base of support in the waist twister. Future intervention may adopt a change of visual input in the training protocol. Participants are healthy community-dwelling adults, so future studies could recruit frail older adults or seniors with specific needs to determine the effectiveness of OFE training. For instance, researchers could recruit seniors with sarcopenia for OFE muscle strength training or rehab seniors with limited ROM for OFE flexibility. Large randomized controlled trials could also provide more definitive evidence. It is also important to consider social factors that influence seniors’ use of OFE, such as facilitators and barriers. 

In sum, there is keen interest in providing proper instructions for OFE use [37]. The contribution from this study is that it provides intervention with ACSM guidelines for older adult OFE exercise. It establishes the groundwork to develop OFE training protocols and exercises. It promotes dissemination of an evidence-based intervention to improve specific fitness goals in terms of objective measurement and functional capacity, which earlier studies endorsed [23,71]. This study highlights research needing more investigation to examine the effectiveness of OFE for populations with special needs.

## Figures and Tables

**Table 1:** Research Protocol and assessments.

Fitness Goal	Intervention Frequency	OFE	Exercise Prescription	Duration	Research Device		Functional Fitness Test
Cardiorespiratory endurance	5 days/week	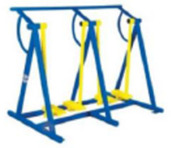 Air walker	Operation tempo 60 bpm	20 min	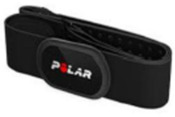 POLAR H10	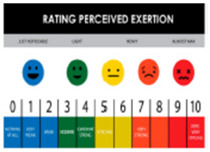 RPE scale	2 min Step
		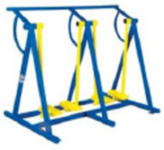 Ski machine		20 min			
Muscle strength	2 days/week	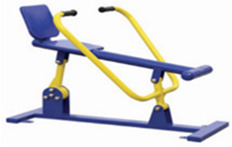 Rowing machine	Week 1–6, 8 reps 3 sets	10 min (1.5 min break between every set)	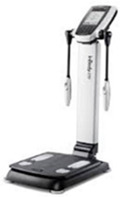 Inbody 270	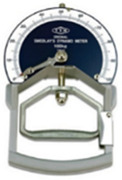 Handgrip dynamometer	30 s Chair stand
		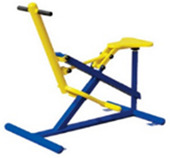 Bonny rider	Week 7–12, 12 reps 3 sets				
Flexibility	2 days/week	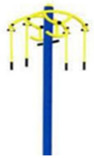 Arm stretch	Per stretching time for 30 s	5 min	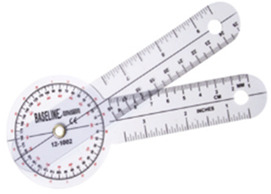 Joint angle protractor		Back Scratch
		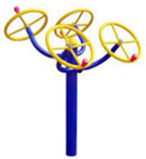 Shoulder wheel	Operation tempo 60 bpm	5 min			
Balance	2 days/week	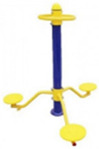 Waist twister	Operation tempo 60 bpm	10 min	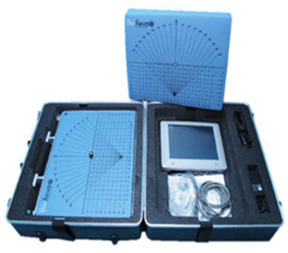 Biosway		Single-leg stand, 8-Foot Up-and-Go
			Week 1–4, 2 feet for 10 min				
			Week 5–8, 2 feet for 10 min, 1 foot for 2 min				
			Week 6–12, 2 feet for 6 min, 1 foot for 4 min				

**Table 2:** Assessments during pre-, mid-, and post OFE intervention.

	Fitness Data		
Test	Pre	Mid	Post
Cardiorespiratory endurance			
2 min step (times)	96.60 ± 2.62 ^bc^	106.90 ± 3.08 ^ac^	115.70 ± 3.07 ^ab^
Muscle strength			
Skeletal muscle weight (kg)	22.00 ± 0.81	21.35 ± 1.15	22.00 ± 0.86
Body fat percentage (%)	29.46 ± 1.28	29.49 ± 1.56	30.14 ± 1.16
SMI (kg/m^2^)	6.53 ± 0.21	6.62 ± 0.20	6.52 ± 0.22
Handgrip strength (kg)	24.52 ± 1.31 ^c^	24.82 ± 1.56 ^c^	27.97 ± 1.61 ^ab^
30 s chair stand (times)	20.72 ± 1.11 ^c^	21.31 ± 1.04 ^c^	24.35 ± 1.17 ^ab^
Flexibility			
Back scratch (cm)	−3.13 ± 2.52	−3.97 ± 2.81	−1.56 ± 2.97
Shoulder horizontal abduction (left) (deg)	52.93 ± 2.44	57.45 ± 1.70	57.76 ± 2.70
Shoulder horizontal abduction (right) (deg)	54.17 ± 2.15 ^b^	63.24 ± 1.90 ^a^	58.66 ± 2.45
Shoulder horizontal adduction (left) (deg)	126.00 ± 4.05	133.55 ± 3.76	133.17 ± 4.59
Shoulder horizontal adduction (right)(deg)	127.66 ± 2.42	129.10 ± 2.79	125.48 ± 4.54
Shoulder internal rotation (left)(deg)	70.45 ± 3.27	67.59 ± 2.95	66.41 ± 3.70
Shoulder internal rotation (right)(deg)	68.72 ± 2.71	67.10 ± 1.98	62.86 ± 2.45
Shoulder external rotation (left)(deg)	90.41 ± 3.17	85.86 ± 2.91	86.07 ± 2.11
Shoulder external rotation (right)(deg)	95.86 ± 2.73	88.79 ± 2.56	89.10 ± 3.86
Shoulder flexion (left)(deg)	169.59 ± 4.47	169.97 ± 1.59	174.86 ± 3.86
Shoulder flexion (right)(deg)	169.52 ± 4.20	166.35 ± 1.49 ^c^	178.38 ± 2.05 ^b^
Shoulder extension left hand (deg)	61.28 ± 2.82	57.24 ± 2.55	56.03 ± 3.06
Shoulder extension right hand (deg)	53.72 ± 3.02	48.83 ± 2.08	52.97 ± 2.82
Shoulder abduction left hand (deg)	140.45 ± 4.41	154.55 ± 2.91	143.90 ± 6.64
Shoulder abduction right hand (deg)	148.03 ± 4.17	156.35 ± 3.30	151.17 ± 4.63
Balance			
8-ft up and go (s)	4.93 ± 0.11	4.92 ± 0.12	4.73 ± 0.10
Single-leg stance (s)	23.84 ± 1.69 ^bc^	27.79 ± 1.07 ^a^	27.80 ± 1.09 ^a^
Eyes open firm surface	0.78 ± 0.06	0.68 ± 0.04	0.73 ± 0.06
Eyes closed firm surface	0.79 ± 0.05	0.75 ± 0.07	0.75 ± 0.04
Eyes open foam surface	1.38 ± 0.07 ^b^	1.16 ± 0.06 ^a^	1.20 ± 0.06
Eyes closed foam surface	2.36 ± 0.15	2.35 ± 0.15	2.10 ± 0.13

^a^ Significant difference from pre-test (*p* ≤ 0.05); ^b^ Significant difference from mid-test (*p* ≤ 0.05); ^c^ Significant difference from post-test (*p* ≤ 0.05).

## Data Availability

The data presented in this study are available on request from the corresponding author.
